# Irisin Induces Apoptosis in Metastatic Prostate Cancer Cells and Inhibits Tumor Growth In Vivo

**DOI:** 10.3390/cancers15154000

**Published:** 2023-08-07

**Authors:** Khalil H. Alshanqiti, Sumayyah F. Alomar, Nourah Alzoman, Aliyah Almomen

**Affiliations:** Department of Pharmaceutical Chemistry, College of Pharmacy, King Saud University, Riyadh 11495, Saudi Arabianalzoman@ksu.edu.sa (N.A.)

**Keywords:** prostate cancer, αVβ5, irisin, apoptosis

## Abstract

**Simple Summary:**

Prostate cancer is the second most common cancer in males worldwide, with αVβ5 integrin being highly expressed in advanced prostate cancer. Irisin, a hormone secreted from skeletal muscles, has been found to reduce the viability and migration of rapidly dividing cells and may have an inhibitory effect on αVβ5. In vitro evaluations showed that irisin reduced PC-3 cell viability to 70%, increased Annexin-V/7AAD positive cell population, altered the expression of apoptotic proteins, and inhibited tumor growth in vivo. This finding can serve as a foundation for further evaluation of irisin’s role in prostate cancer.

**Abstract:**

Background: Prostate cancer is the second most common cancer in males worldwide, with αVβ5 in-tegrin, a coactivator receptor, being highly expressed in advanced prostate cancer. Irisin, a hormone secreted from skeletal muscles, can reduce cell viability and migration and potentially inhibit αVβ5. Objective: This study investigates the potential impact of irisin on prostate cancer cells and its underlying mechanism. Methods: In vitro evaluation of the antiproliferative action of irisin on metastatic prostate cancer (PC-3) cells was tested through MTT assay, flow cytometry, and Western blot. An in vivo evaluation of the antiproliferative effect on prostate cancer xenograft was evaluated in nude mice. Results: In vitro evaluations showed that irisin reduced PC-3 cell viability to 70% and increased the Annexin-V/7AAD positive cell population. Irisin altered the expression of apoptotic proteins, αVβ5, and proteins involved in the P13k-Akt pathway. In vivo, irisin inhibited tumor growth and progression, positively affecting animal well-being. In conclusion, irisin has an apoptotic effect on PC-3, possibly through altering αVβ5 and the Bcl2/BAX and P13k-Akt signaling pathway, inhibiting tumor growth in vivo. Conclusion: Our findings can serve as a foundation for further evaluation of irisin’s role in prostate cancer.

## 1. Introduction

Prostate cancer (PC) is men’s second most frequent malignancy worldwide. PC can be asymptomatic in the early stages and may require minimal or no treatment [1,2]. However, patients often complain of dysuria, increased frequency, and nocturia, which might also occur with benign prostatic hyperplasia [3]. Advanced stages of PC can result in back pain due to tumor metastasis to the axial skeleton, the most common site of metastasis [4,5]. Several factors can contribute to the incidence of PC, such as genetic susceptibility; exposure to unknown external risk factors; or artificial reasons, such as differences in cancer registration and medical services [6]. For example, rates in China and Japan showed lower numbers compared with the United States. In the United States, African Americans showed the highest rates among other races, with about 137 new cases per 100,000 people yearly [4]. Besides genetics, this could be due to differences in lifestyle and diet [5]. It is speculated that prostate cancer will be the most common cancer in men in the upcoming years [4].

According to numerous studies, moderate physical activity can improve the quality of life by reducing the risk of psychological depression, metabolic diseases, certain types of cancer, and chronic illnesses [7,8]. Clinical evidence is mounting to support the claim that physical activity reduces PC risk and increases related survival [9]. This could be driven by the modulation of hormonal secretions such as insulin, growth hormone, cortisol, and irisin [10]. Irisin is a hormone produced from fibronectin type III domain-containing protein 5 (FNDC5), a type I transmembrane protein found in skeletal muscles and other organs [11]. Exercise causes a portion of the FNDC5 protein to be broken and released into the circulation as irisin [11]. Irisin has beneficial effects on humans, which include the induction of thermogenesis and calorie burning [12]. Moreover, irisin induces white adipose tissue browning, mitochondrial uncoupling protein 1 (UCP1)-mediated heat production, and power consumption [12]. Other recent studies on irisin’s mechanism reported that irisin reduces the viability and migration of rapidly dividing cells. Irisin is remarkable for its 100% similarity in humans, rats, and mice [13]. This degree of similarity is much higher than that of other hormones, such as insulin, glucagon, and leptin, which are only 85%, 90%, and 83% similar, respectively [13]. The discovery of this hormone has motivated further research into its potential uses, particularly in terms of human health [13].

In PC, tumor cells are surrounded by significantly abnormal extracellular matrix (ECM) changes expressing an abnormal integrin repertoire [14]. Such changes result in significant consequences, enabling integrin to regulate specific cell functions [14]. αVβ5 is a type of integrin receptor that binds to matrix macromolecules and proteinases, stimulating angiogenesis [15]. Studies have suggested that integrin αVβ5, a cell surface receptor that binds to extracellular matrix proteins, has elevated levels in several cancer types, including prostate cancer [16,17]. The αVβ5 integrin receptor is a coactivator receptor that works along with αVβ3 in the activation process of the growth factor receptor in PC [18]. According to studies, high expression of αVβ5 has been linked to aggressive prostate cancer and worsened patient outcomes [18,19]. αVβ5 has also been demonstrated to facilitate PC tumor cell invasion, migration, and angiogenesis. The mechanism of irisin interaction with αVβ5 remains unclear, yet studies have reported that it is possibly through a biophysical interaction between irisin and αVβ5 surfaces [20]. Therefore, it has been suggested that targeting αVβ5 could be a possible new treatment approach for PC. In this research, the role of irisin in managing PC was evaluated in vitro and in vivo, and the potential downstream signaling pathway involved in this process was tested.

## 2. Materials and Methods

### 2.1. Materials

Irisin was purchased from (Caymanchem, Ann Arbor, MI, USA, Item No. 11451CAS No. 9037-90-5). Caspase 3 (Cat# 9662), BCL-2 (Cat# 124), BAX (Cat# 2D2), Cleaved Caspase 3 (Cat# Asp 175), PARP (Cat# 9532), PI3k (Cat# 17366), AKT (Cat# 4060), BCL-XL (Cat# 2764), and β-Actin (Cat# 93473) were purchased from Cell signaling, Danvers, MA, USA. αVβ5 (Cat# bs 1310 R) was purchased from BIOSS, Woburn, MA, USA.

### 2.2. Cell Culturing

Grade IV prostate cancer cell line (PC-3), catalog number CRL-3470, and normal embryonic kidney epithelial cell line (HEK-293), catalog number CRL-1573 cells were obtained from the American Type Culture Collection (ATCC). PC-3 cells were maintained in Dulbecco’s modified Eagle’s medium (DMEM—Thermo Fisher Scientific, Waltham, MA, USA, Catalogue number: 12491023) containing 10% fetal bovine serum (FBS) (Thermo Fisher Scientific, Catalogue number: 12483020), penicillin–streptomycin (Thermo Fisher Scientific, Catalogue number: 15140148), L-glutamine (Thermo Fisher Scientific, Catalog number: A2916801), and pyruvate sodium (Thermo Fisher Scientific, catalogue number: 11360088). Cells were grown at 37 °C and 5% CO_2_ [21]. The cells were split at 70–80% confluence level.

### 2.3. MTT Assay

A 96-well plate was used to disperse and seed PC-3 or HEK-293 cells at a density of 5 × 10^3^ cells per milliliter. Irisin treatments at various doses were applied to the cells. At various times, the media were removed and replaced with new media. Cells were then treated with 20 L of 2.5 mg/mL 3-(4,5-dimethylthiazol-2-yl)-2,5-diphenyltetrazolium bromide (MTT) (Invitrogen, Bend, OR, USA) in PBS before being cultured for an additional 4 h at 37 °C. After that, 100 L of DMSO was added to dissolve the formazan crystals. A Spectramax 250 microplate reader (Molecular Device, San Jose, CA, USA) was used to detect absorbance at 540 nm. The optical density (OD) (treated cells/OD of control cells) was multiplied by 100 to compute the percentage of cell viability (%).

### 2.4. Flow Cytometry

Apoptotic potential for irisin against PC-3 was evaluated using flow cytometry. PC-3 cells were treated with ≈2 × IC50 (100 nmol/L) irisin for 24, 48, and 72 h. Cells were then harvested, suspended in 500 μL Annexin binding buffer (ABB), and either incubated with 5 μL/mL Annexin-APC (Annexin-V conjugated to allophycocyanin, Life Technologies, Grand Island, NY, USA) for 15 min or stained by adding 1 μL/mL of 7-aminoactinomycin D (7-AAD) Life Technologies (Grand Island, NY, USA) and incubated in ice for 45 min. Treated samples and controls were analyzed using FACSCanto-II (BD-BioSciences, San Jose, CA, USA) at the College of Pharmacy, King Saud University. APC was excited with a 635 nm laser and detected at 660 nm for Annexin-V and with a 488 nm laser and detected at 660 nm for 7-AAD Assay.

### 2.5. Western Blot

PC-3 cells were plated in 10 cm^2^ culture dishes; treated with irisin for 24, 48, and 72 h; and then analyzed using a Western blot. Cell lysate was prepared using a radioimmunoprecipitation assay (RIPA) buffer (Bio-Rad, Hercules, CA, USA). The amount of protein in the cell lysate was calculated using the DCTM Protein Assay Kit (Bio-Rad, Hercules, CA, USA). Twenty microliter/well of irisin-treated and control cells’ lysates were loaded into 4–20% Mini-Protean TGX Gels (Bio-Rad, Hercules, CA, USA), subjected to electrophoresis, and then transferred to a PVDF membrane. Membranes were blocked with 5% (*w*/*v*) skim milk in Tris-buffered saline and Tween 20 (TBS-T) and then incubated with primary antibodies overnight at 4 °C according to the manufacturer’s instructions. Following a wash, membranes were treated with the appropriate secondary antibody and bands were detected using the Western Bright ECL Kit for 5000 cm^2^ Membrane and Blue Basic Autoradiography Film (Bioexpress, Kaysville, CA, USA).

### 2.6. Animals

Twelve Nu/Nu mice, six-to-eight-week-old males weighing 22–28, were obtained from the animal central lab, King Saud University, Riyadh, Saudi Arabia. Animals were kept on a 12/12 h light/dark cycle and housed at a temperature of 20–25 °C and humidity of about 40%. Animal experiments adhered to the guidelines of the Ethical Committee for Performing Studies on Animals, King Saud University, Riyadh, Saudi Arabia, following protocol KSU-SE-22-106.

### 2.7. In Vivo Evaluation of Irisin in Prostate Cancer Xenograft Mouse Model

Five million PC-3 cells in 100 μL Matrigel made in serum-free medium were injected subcutaneously into the lower-right flank of mice. Twenty days after cell suspension was injected and the tumor became apparent, animals were randomized into either docetaxel (*n* = 4) as treatment control, irisin-treated group (*n* = 4), and non-treated control (*n* = 4).

Peritumoral injection of 100 μL of (1 mg/mL) irisin or docetaxel (dissolved in sterile water for injection), or 100 μL of sterile water for injection (control), was carried out three times a week for 21 days. Tumor volumes were measured using the following formula: V = 0.5 (L × W2), where (L) is the length and (W) is the width of the tumor. Animals were sacrificed one day after the last injection, and tumors and heart tissue were resected. Tissues were dehydrated in 70% ethanol after being fixed in 10% formalin for 48 h. Using light microscopy, two different pathologists independently examined the Hematoxylin and Eosin (H&E) stains on representative tissues.

## 3. Results

### 3.1. Irisin Impairs the Viability of PC-3 Cells and Maintains the Viability of Normal Epithelial Cells

**Figure 1:**
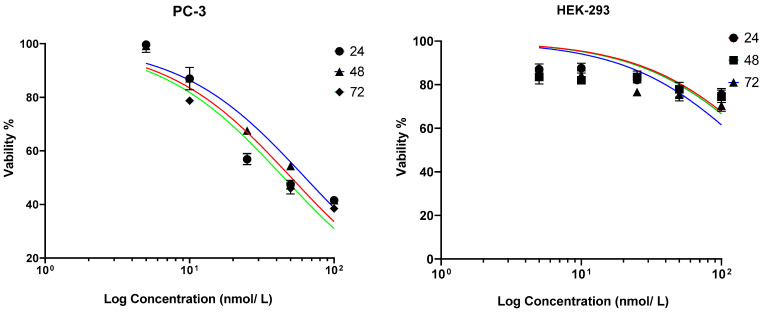
Irisin impairs the viability of PC-3 cells and maintains the viability of normal epithelial cells. Dose–response curve for PC-3 cells (**left**) and HEK-293 cells (**right**).

The effect of irisin on PC-3 cell viability at multiple concentrations of irisin (5, 10, 25, 50, and 100 nmol/L) for 24, 48, and 72 h are shown in Figure 1. Results show that PC-3 is sensitive to irisin and exhibits a dose-dependent decrease in cell viability apparent at 24 h post-treatment, reaching about 38% viability only with 100 nmol/L at 72 h. IC-50s at 24, 48, and 72 h were 50.59, 63.17, and 44.90 nmol/L, respectively, in PC-3 cells. In Hek-293 cells, viability was mostly about 70% with 100 nmol/L, the highest concentration of irisin used in this study.

### 3.2. Irisin Boosts Annexin-V and 7-AAD Positive Cell Numbers and Induces Apoptotis

**Figure 2:**
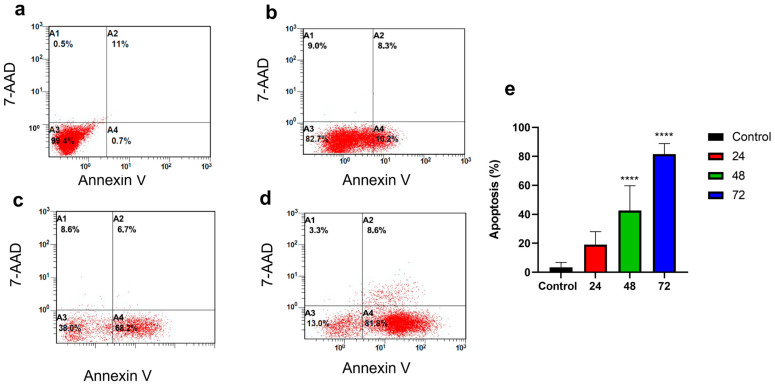
Irisin increased the percentage of PC-3 apoptotic cells relative to control (**a**) compared to 24 h (**b**), 48 h (**c**), and 72 h (**d**). Increase if % apoptosis was about 5.5-, 12.2-, 23.34-fold relative to control at 24, 48, and 72 h, respectively (**e**). Statistical significance was obtained with *p*-values ≤ 0.05, and **** *p* < 0.0001.

To confirm if irisin can induce apoptosis in prostate cancer through flow cytometry, both 7-AAD and Annexin-V were used. Figure 2 shows that compared with untreated cells, PC-3 cells increased in 7-AAD and Annexin-V positive cell populations after irisin treatment. The increase in % apoptosis increased by about 5.5-, 12.2-, and 23.34-fold relative to control at 24, 48, and 72 h, respectively (Figure 2e).

### 3.3. Irisin Alters the Expression of Apoptotic Proteins

**Figure 3:**
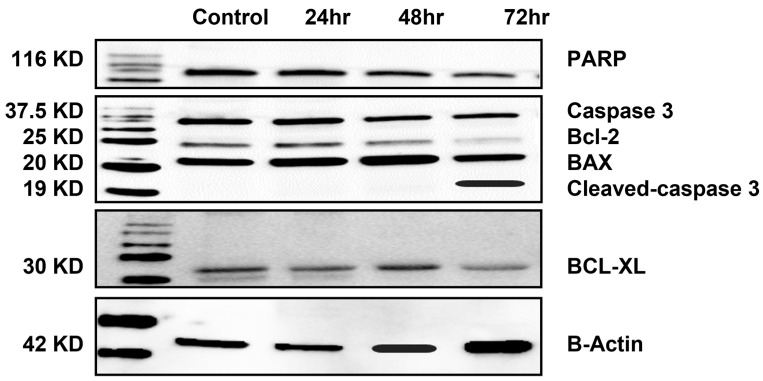
Irisin alters the expression of the apoptotic protein. Western blot for PARP, Caspase3, cleaved caspase 3, Bcl-2, BAX, and BCL-XL after 24, 48, and 72 h of irisin treatment. B-Actine was used as a loading control. The uncropped blots are shown in File S1.

To confirm the apoptotic potential of irisin in PC-3 cells, apoptotic protein expression was evaluated after the exposure of the cell to ≈IC50 (50 nmol/mL) of irisin for 24, 48, and 72 h. Results in Figure 3 and Figure S2 show that irisin was able to activate caspase 3, exhibited by the increase in the expression of cleaved caspase 3 levels, which further induced cleavage of PARP. Proteins involved in the intrinsic apoptotic pathway—Bcl-2, Bcl-2 homolog B-cell lymphoma extra-large (Bcl-xL), and the pro-apoptotic protein BAX—were also evaluated after irisin treatment. Although irisin was able to decrease expression levels of Bcl-2 and Bcl-XL over time, the expression level of BAX was not affected.

### 3.4. Expression Levels of αVβ5, PI3K, and AKT in PC-3 Cells Are Downregulated by Irisin

**Figure 4:**
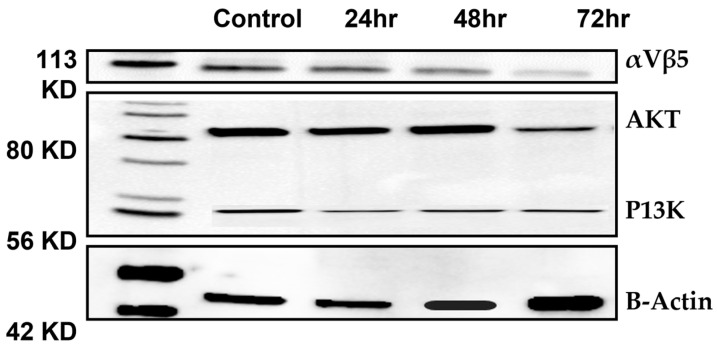
Expression levels of αVβ5, PI3K, and AKT in PC-3 cells are by irisin. Western blot for αVβ5, PI3K, and AKT after 24, 48, and 72 h of irisin treatment. B-Actine was used as a loading control. The uncropped blots are shown in File S1.

To evaluate the role of irisin in inhibiting cancer cell proliferation via inhibiting integrin, expression levels of αVβ5, PI3K, and AKT were evaluated. Figure 4 and Figure S2 indicate that expression levels of αVβ5 and P13K were decreased as early as 24 h post irisin treatment. However, the decrease in Akt expression took more time and was apparent at 72 h post-treatment.

### 3.5. Irisin Inhibits Tumor Growth in Prostate Cancer Xenograft

**Figure 5:**
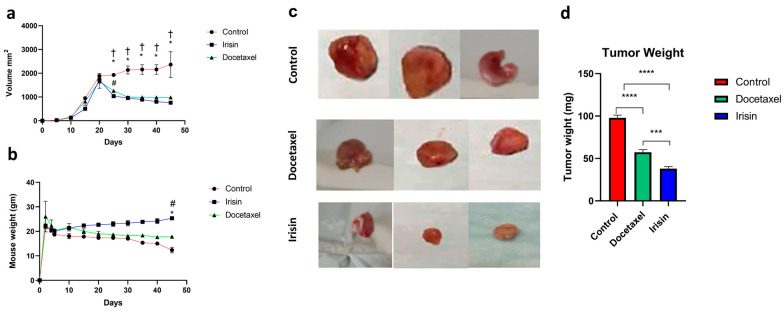
Irisin inhibited tumor growth in prostate cancer xenografts. (**a**) Tumor volume of the treated group compared with docetaxel and control (tumor treatment started at day 20 post cell suspension injection in mice). (**b**) Mice body weight throughout treatment. (**c**) Representative figure of excised tumors from different treatment groups and (**d**) excised tumor weights. * Indicates statistical significance between the irisin and control group and † indicates statistical significance between the docetaxel and control group. # Indicates statistical significance between irisin and docetaxel group. *p*-values of <0.05 were considered statistically significance, where *** *p* < 0.001, and **** *p* < 0.0001.

To evaluate the safety and efficacy of irisin in managing prostate cancer in vivo, animals were treated three times a week for 21 days with either irisin, docetaxel, or vehicle. During the 21 days of irisin treatment, tumor growth and progression were attenuated relative to control (Figure 5a). It was also observed that irisin tumor growth inhibition was almost similar to that of docetaxel. However, there was a significant difference in animal weight between animals treated with irisin and docetaxel and those treated with irisin and control during therapy (Figure 5b).

Histopathological evaluation of cardiac tissues shows that cardiac tissue in the controlled non-treated group had a loss of myofibrillar pattern, cytoplasmic vacuolation, and disorganization of myocardial muscle, a pattern of dilated cardiomyopathy (Figure S1a). In the docetaxel-treated group, cardiac tissues showed disorganization of myocardial muscle fibers and myofibrillar loss with no apparent nuclear degenerative changes or cytoplasmic vacuolation (Figure S1b). However, cardiac tissues of animals treated with irisin showed the typical architecture of cardiomyocytes and connective tissue (Figure S1c). Moreover, the size and weight of the tumor excised at the end of the 21 days of treatment in the irisin-treated group were significantly different from those of the docetaxel group (Figure 5c,d).

## 4. Discussion

Prostate cancer, the second most common cancer among men, varies in incidence by race and country, possibly due to genetics, lifestyle, and diet [1,4]. While early-stage prostate cancer can be asymptomatic, advanced stages may cause dysuria, increased frequency, nocturia, and back pain [4,5]. The treatment of choice for metastatic prostate cancer is docetaxel monotherapy [22]. Although docetaxel can be tolerated by patients, effects such as fatigue, reaction at the infusion site, pneumonitis, fluid retention, and febrile neutropenia can affect the well-being of the patients [23].

Physical activity improves the quality of life and prevents chronic diseases, metabolic diseases, depression, and certain types of cancers [8,9]. Irisin, a hormone secreted from skeletal muscles during exercise, is thought to have beneficial effects, such as heating the body and burning calories [13]. Studies show that irisin might reduce the viability and migration of rapidly dividing cells; however, its role in prostate cancer remains unclear [24,25]. Irisin’s mechanism in cancer is not fully understood, but it may interact with αVβ5 integrin receptors, which play a role in angiogenesis and growth factor activation in prostate cancer [15,18,20]. Therefore, it was interesting to find the role of irisin on metastatic prostate cancer cells and evaluate if it can be a safer therapeutic alternative for managing prostate cancer.

The results demonstrated that PC-3 cells were sensitive to irisin treatment, exhibiting a dose-dependent decrease in cell viability over time (Figure 1). However, at 48 h of irisin-treated cells’ incubation, cells were trying to resist the cytotoxic effect of irisin, apparent by the increase in IC-50. However, cells lost the ability to resist a decrease in IC-50 at 72 h post-treatment. The IC-50 values for PC-3 cells at 24, 48, and 72 h were 50.59, 63.17, and 44.90, respectively. Interestingly, irisin did not show a drastic change in normal epithelial cell viability, which was maintained at about 70%, even with the highest dose of irisin used in this study.

Since one of the ultimate goals of cancer therapy is to induce apoptosis, the increase in Annexin-V and 7-AAD cell populations after irisin therapy was evaluated. Although there was no significant increase in % apoptosis after 24 h of treatment, it seems that cells were dormant before going through apoptosis, as shown by the significant increase in % apoptosis at the later times of 48 and 72 h. These results are in parallel with Western blot data, where the change in PARP-cleaved caspase-3 was mostly clear at 72 h post-treatment.

Recent studies have suggested that inhibition of αVβ5 integrin may affect the expression of the anti-apoptotic protein Bcl-2 in cancer cells [26]. Bcl-2 is a member of the Bcl-2 family of proteins, which are essential components in regulating apoptosis or programmed cell death [27]. Bcl-2 proteins achieve this by controlling the permeability of the mitochondrial outer membrane. The inhibition of αVβ5 integrin could potentially alter the expression of Bcl-2, thereby influencing apoptosis [28]. Our data also show that irisin can activate that intrinsic apoptosis pathway found with decreased Bcl-2 and BCL-XL expression and the disturbance of the Bcl-2/BAX ratio [28].

Integrins are an essential class of transmembrane glycoproteins that play a critical role in cell adhesion, motility, proliferation, and differentiation. Integrins are composed of two subunits, the α and β subunits, and each subunit has three distinct domains: extracellular, transmembrane, and cytoplasmic domain [29]. While the extracellular domain binds to adhesive proteins, growth factors, and other ligands in the extracellular matrix (ECM), the transmembrane domain anchors integrin to the plasma membrane [29]. The cytoplasmic domain activates and regulates downstream signaling pathways essential for cell invasion, angiogenesis, and other processes [29]. It is critical to stress that each of these integrin functions support the ability of tumor cells to metastasize [29].

Recent studies on the αVβ5 integrin have examined its potential role in cancer treatment. Evidence suggests that the αVβ5 integrin can activate the PI3K/AKT pathway in cancer cells, increasing cell survival and proliferation. Therefore, targeting the αVβ5 integrin may provide a therapeutic opportunity for treating cancer [30,31]. Our results showed the ability of irisin to downregulate the expression of αVβ5, which probably altered the AKT/PI3K signaling pathway.

One of this study’s goals is to evaluate irisin’s safety and efficacy in treating prostate cancer in vivo. Although our results showed a significant difference in tumor size after treating the tumor with irisin, docetaxel was able to shrink the mouse’s tumor similarly. Unlike docetaxel, which caused weight loss in mice, irisin appeared to maintain animal weight, which can be considered a sign of well-being. Moreover, histopathological evaluation showed that irisin is safe for cardiac tissue, further highlighting its promise as a more tolerable treatment for PC. Signs of cardiac myopathy seen in the control group can be explained by previous studies indicating that cancer or aberrant cellular development elsewhere in the body can cause cardiac dysfunction through rising ROS levels throughout the body [32].

## 5. Conclusions

Irisin shows an apoptotic effect on PC-3, probably through the alteration of αVβ5 and, thus, the Bcl-2/Bax and P13k-Akt signaling pathways, as presented in Figure S3. Irisin also inhibited tumor growth in vivo with no apparent toxicity. Our results can be used to further investigate irisin’s role in prostate cancer.

## Data Availability

Data are contained within the article or Supplementary Materials.

## Supplementary Materials

The following supporting information can be downloaded at: https://www.mdpi.com/article/10.3390/cancers15154000/s1, Figure S1: Histological evaluation of mice cardiac tissue of (a) Control group, animals bearing prostate cancer with no treatment (H&E stain, magnification 40×), (b) Docetaxel treated mice group (H&E stain, magnification 40×), and Irisin treated group (H&E stain, magnification 40×). Figure S2: Quantification of western blot band density. Figure S3: A possible pathway for irisin inducing of apoptosis in prostate cancer cells. File S1: Full pictures of the Western blots.
