# Supplementary material for: Irisin Induces Apoptosis in Metastatic Prostate Cancer Cells and Inhibits Tumor Growth In Vivo
Source: Cancers (Basel). 2023 Aug 7;15(15):4000. doi: 10.3390/cancers15154000 (PMC10416853; doi:10.3390/cancers15154000)
Supplement: Supplementary file 1 [file cancers-15-04000-s001.zip › cancers-2517393-Figures S1-S3.docx]

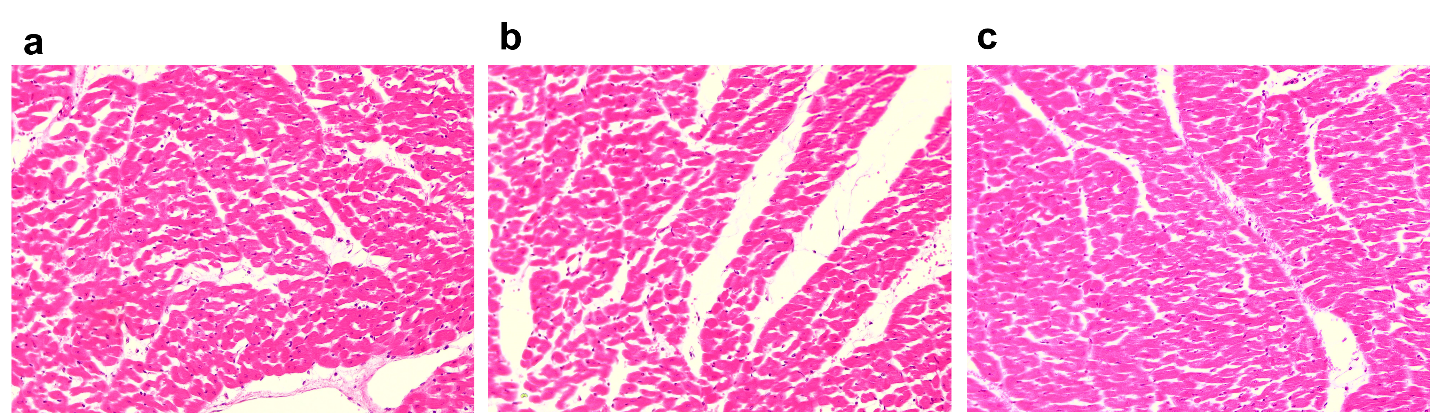


**Figure S1.** Histological evaluation of mice cardiac tissue of a) Control group, animals bearing prostate cancer with no treatment (H&E stain, magnification 40X), b) Docetaxel treated mice group (H&E stain, magnification 40x), and Irisin treated group (H&E stain, magnification 40x).

**Figure S2.** Quantification of western blot band density.


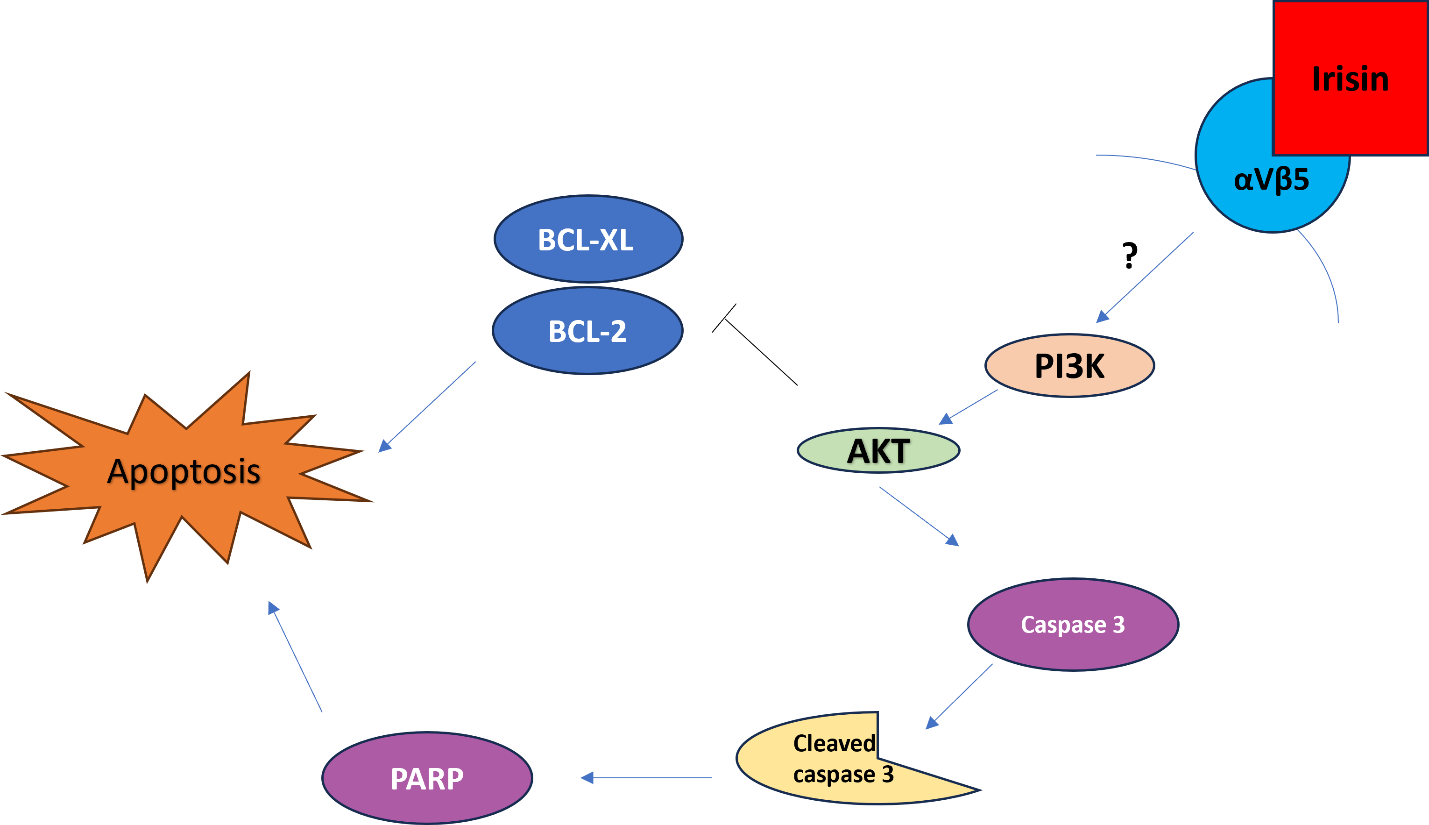


**Figure S3.** A possible pathway for irisin inducing of apoptosis in prostate cancer cells.
