# Supplementary figures and images for: Irisin Induces Apoptosis in Metastatic Prostate Cancer Cells and Inhibits Tumor Growth In Vivo
Source: Cancers (Basel). 2023 Aug 7;15(15):4000. doi: 10.3390/cancers15154000 (PMC10416853; doi:10.3390/cancers15154000)

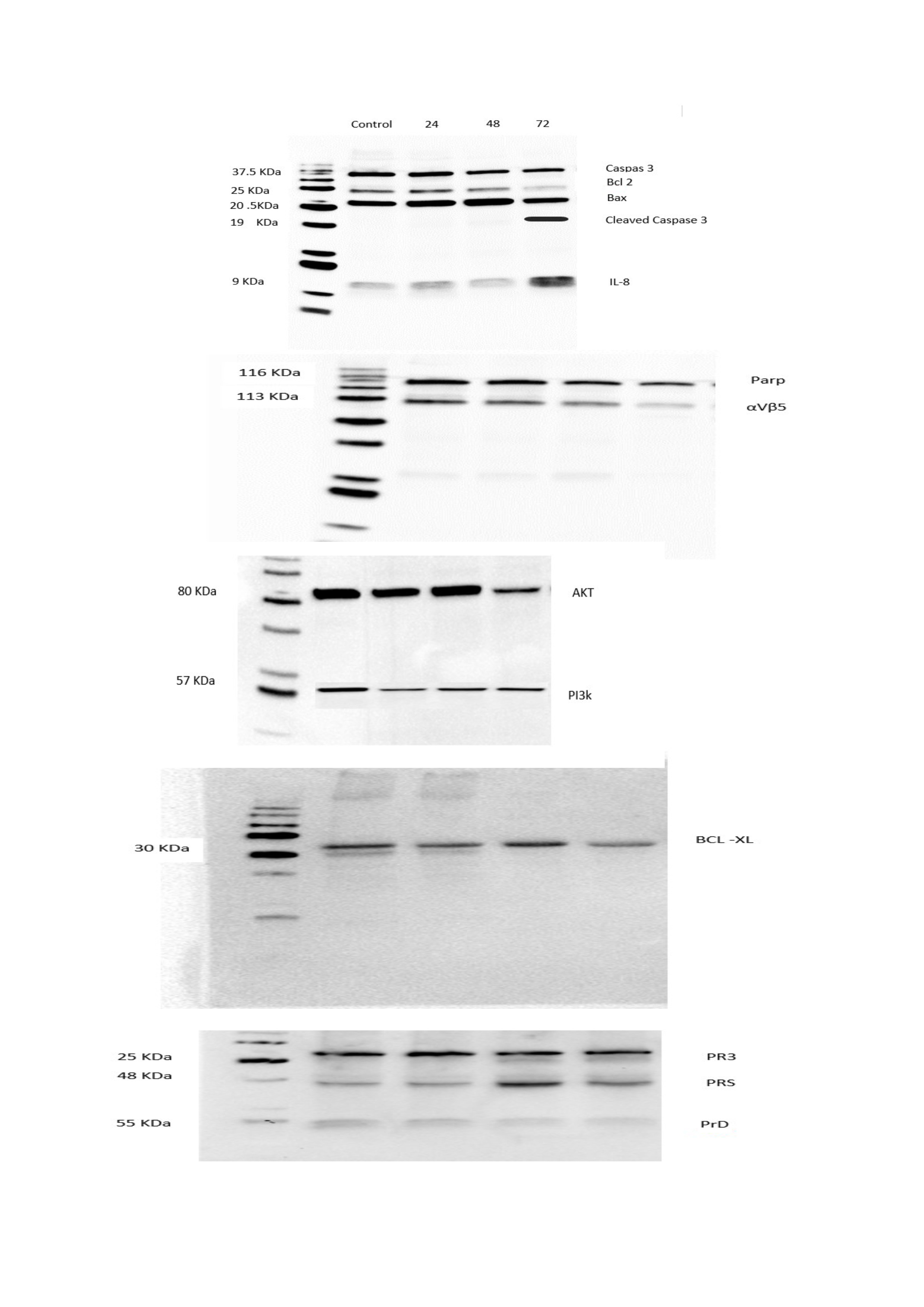


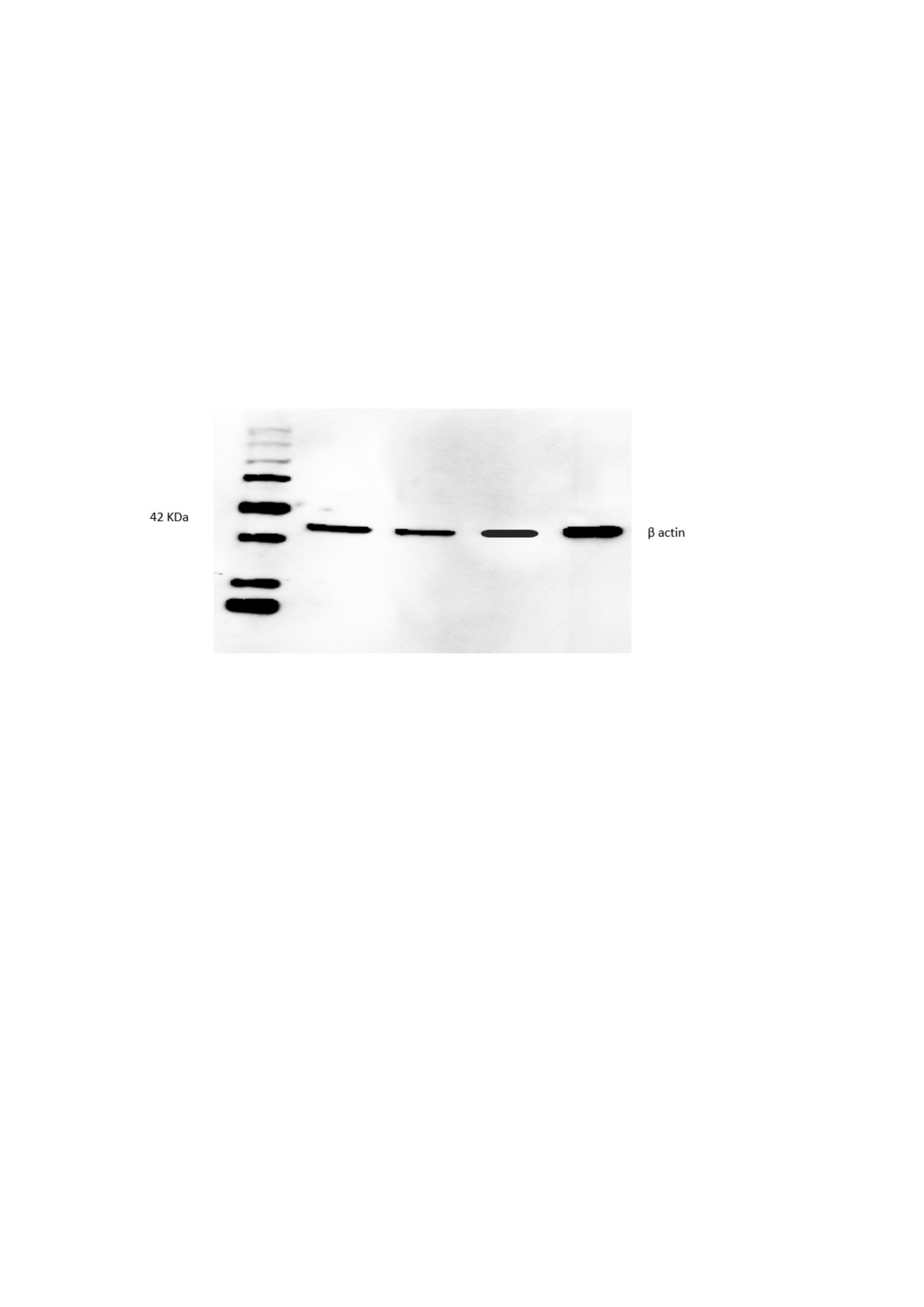

Supplement: Supplementary file 1 [file cancers-15-04000-s001.zip › cancers-2517393-File S1.docx]
